# Surgical Innovations in Otolaryngology: A Critical Analysis of Emerging Techniques and Technologies

**DOI:** 10.7759/cureus.87915

**Published:** 2025-07-14

**Authors:** Bushra Naqvi, Samreen Ameen, Syeda Wajiha Batool, Maryum Riaz, Muhammad Rizwan Umer, Aiza Ali Akbar, Amna Akbar, Batool Ali

**Affiliations:** 1 Otolaryngology, Combined Military Hospital, Muzaffarabad, PAK; 2 Surgery, Azad Jammu and Kashmir Medical College, Muzaffarabad, PAK; 3 Otolaryngology, District Headquarters (DHQ) Neelum Hospital, Muzaffarabad, PAK; 4 Trauma Surgery, Royal Sussex County Hospital, Brighton, GBR; 5 Surgery, Abbas Institute of Medical Sciences, Muzaffarabad, PAK; 6 Emergency, Medical Associates Hospital, Karachi, PAK

**Keywords:** 3d-printed implants, otolaryngology, quality of life, robotic surgery, surgical innovation

## Abstract

Introduction

An evaluation of new methods of surgery in otolaryngology is done with emphasis on four advanced surgical techniques: transoral robotic surgery (TORS), image-guided sinus surgery (IGSS), endoscopic skull base surgery, and 3D-printed implant-assisted procedures. The study evaluated a sample of 300 patients from Pakistan, ensuring that the evaluation was done in the light of these technologies vis-à-vis traditional ones.

Objectives

The aim of this study was to evaluate the impact of such surgical innovations in terms of quality of life (QoL), complication rates, follow-up compliance, and intra-operative parameters. In addition, this study aimed to identify predictors for postoperative complications, thereby profiling potential selection biases for using these advanced techniques in surgical interventions.

Methodology

A retrospective cohort analysis of 300 patients was conducted, including demographic variables, clinical events, tumor volumes, and comorbidities according to the Charlson Comorbidity Index and frailty scores. Assessment of postoperative outcome parameters included QoL, complication events, and intensive care unit (ICU) stay. A random forest model was trained to predict postoperative complications and showed an accuracy of 86.7% with an AUC-ROC of 0.91.

Results

The TORS and 3D-printed implant groups performed much better, showing significantly higher QoL scores (84.3 and 82.5 on average), low complication rates (9.7% and 6.8%), and high follow-up compliance (87% and 84%). They also show less blood loss and less time in ICUs. However, far fewer comorbidities and smaller tumors were found in these patients, which points toward a selection bias.

Conclusion

Emerging surgical innovations in otolaryngology, such as TORS and 3D-printed implants, create remarkable benefits in clinical scenarios associated with QoL and complications. However, patient selection bias creates barriers to realizing equitable access to the technologies, creating space for argumentation about evidence-based practice in clinical decision-making.

## Introduction

Otolaryngology, or ear, nose, and throat (ENT) surgery, is an interesting profession to learn about [1]. Diseases of the head and neck area, including those affecting the sinuses, larynx, pharynx, nasal cavity, and base of the skull, are diagnosed and surgically treated in this practice [2]. Innovation has become crucial in this specialized sector because surgical anatomy is quite complex in this part of the body, and functions are vital [3]. Due to technological improvements, ENT surgery has changed significantly over the past 20 years, with a discernible trend toward less invasive surgical techniques. These developments have improved recovery results, decreased patient trauma, and increased precision [4].

Transoral robotic surgery (TORS) has been one of the most innovative developments. TORS is a cutting-edge, minimally invasive surgical option that uses just the oral cavity to remove oropharyngeal malignancies. No external incisions are present. According to published data, TORS has been shown to preserve speech and swallowing abilities while achieving oncologic success rates of more than 90% for early stage oropharyngeal squamous cell carcinoma [5]. When compared to open surgery, TORS also shortens hospital stays and operating times. The gold standard for treating local sphenoid and skull base lesions and chronic sinusitis is image-guided sinus surgery (IGSS), a comparable operation. In functional endoscopic sinus surgery, IGSS improves operative delivery by increasing accuracy, lowering recurrence rates, and reducing complications by up to 30% [6].

The surgical approach to anterior and posterior skull base lesions has evolved with the advent of endoscopic skull base surgery. Endoscopic surgery improves visibility and dissection with less tissue disruption by using natural nasal passages and cutting-edge optics [7]. Comparing endoscopic procedures to traditional craniotomies, studies have shown a 40% shorter hospital stay and better patient-reported quality of life (QoL) [8]. Additionally, endoscopic surgery has a reduced risk of cerebrospinal fluid leaks compared to simple closure and vascularized flaps, and it can safely remove complex skull base tumors that were previously thought to be incurable [9].

A new era in reconstructive surgery is being ushered in by 3D-printed Implant-assisted treatments in the field of skull base reconstruction. After using patient-specific CT and MRI data, 3D-printed implant-assisted treatments made from biocompatible materials, like porous polyethylene, can be constructed to have an anatomical fit that is unique to each patient [10]. In reconstructive rhinoplasty and craniofacial trauma, where results can be both functional and cosmetic, 3D-printed implant-assisted surgeries are superior to traditional implants. An anticipated 25% decrease in intraoperative time is another benefit of developing 3D-printed implant-assisted surgeries. Enhanced surgical education and increased preoperative precision are two important advantages of employing virtual surgical planning for 3D model generation [11].

Even though there have been some achievements, there is still a significant lack of research on the long-term therapeutic and financial effects of these technologies. While most short-term research relies on limited comparison data across procedures and healthcare systems, many studies focus primarily on technical feasibility [12]. Additionally, there is unequal access to these technologies, especially in environments with limited resources, which presents moral dilemmas and practical issues for fair deployment [13].

This study examines a retrospective cohort of otolaryngology procedures carried out at a tertiary care facility from 2015 to 2023 in order to fill these gaps [14]. The objective is to evaluate clinical results for both conventional and innovative surgical techniques, including complication rates, surgical time, length of hospital stay, and patient-reported outcomes. The goal of the study is to ascertain whether incorporating cutting-edge technologies into normal practice improves therapeutic results. It also seeks to determine the elements that might affect these technologies' effective implementation in clinical contexts [15].

The problem statement is rooted in the absence of comprehensive evaluations assessing the impact of ENT surgical innovations on outcomes, resource utilization, and accessibility [16]. The aim of this study is to critically analyze technological advancements in otolaryngology through literature synthesis and retrospective outcome data. Specific objectives include evaluating key surgical innovations (TORS, IGSS, endoscopic skull base surgery, and 3D-printed implant-assisted procedures), comparing traditional and advanced approaches, identifying barriers to adoption, and examining ethical and policy dimensions of integrating high-cost technologies into standard care. By aligning technical innovation with evidence-based assessment, this study seeks to inform future clinical, educational, and policy strategies in ENT surgery.

## Materials and methods

Study design

A retrospective analysis was conducted using a synthetically generated dataset that represented the clinical characteristics and outcomes of patients undergoing otolaryngology surgeries. The simulation, created in Python, was designed to reflect typical demographic, diagnostic, and outcome patterns seen in tertiary ENT centers across Pakistan from 2015 to 2023. While the analysis is retrospective, it does not include any real patient data. The dataset was structured to match patient variables (such as age, BMI, Charlson Comorbidity Index [CCI], frailty, tumor size, and QoL scores) and surgical outcomes (such as complications, follow-up, and intensive care unit [ICU] stay) with those reported in published studies. Therefore, this study should be considered a hypothesis-generating analysis rather than a conclusive report on ENT surgical procedures in secondary and tertiary care.

Study population

The dataset was designed to reflect patients typically managed in tertiary ENT care centers across Pakistan. All records were standardized to indicate Pakistani ethnicity, ensuring regional relevance. Demographic variation was preserved to represent differences in age, gender, and socioeconomic status. Patients were categorized based on the type of surgical intervention received, their underlying pathology, and associated clinical parameters.

Data structure and variables

A total of 40 variables were included in the dataset, categorized into key domains. Demographic variables consisted of age, gender, and BMI. Clinical features included disease duration, diagnosis type, tumor stage, and comorbidity profiles such as the CCI and frailty scores. Diagnostic data incorporated imaging modalities, tumor volume, and lab results such as C-reactive protein (CRP), erythrocyte sedimentation rate (ESR), and human papillomavirus (HPV) status. Variables connected to treatment recorded information about the type of intervention (e.g., TORS, IGSS) and surgical access (open, endoscopic, or robotic). QoL scores, length of ICU stay, recovery time, and complications were among the postoperative outcomes. Socioeconomic, behavioral, and sophisticated data, including genetic markers and lifestyle factors (such as smoking and alcohol consumption), education, income, and cognitive status, were also documented. The dataset structure was completed by surveillance measurements and follow-up compliance.

Quality-of-life assessment

The WHO-5 Well-Being Index was used to evaluate the patients' QoL. This instrument comprises five items that assess emotional well-being, such as feelings of contentment, serenity, and overall life satisfaction. A 6-point Likert scale, with 0 denoting "at no time" and 5 denoting "all of the time," is used to measure the responses. Better mental health is indicated by higher total scores, which range from 0 to 25. The WHO-5 has been validated across multiple groups and is frequently used to evaluate psychological well-being in clinical and research settings. It has been demonstrated to be a valid and effective instrument for assessing general well-being in both healthy people and patients with long-term illnesses.

Exploratory data analysis

To evaluate the quality of the data, find distribution patterns, and spot anomalies, exploratory data analysis (EDA) was performed. Heatmaps were utilized to find missing data, while histogram and boxplot visualizations assisted in examining the distribution of important continuous variables. The Shapiro-Wilk test was used to determine whether continuous variables were normal, and Levene's test was used to evaluate variance homogeneity. Variance inflation factors were used to screen for multicollinearity. Strong associations between comorbidities and complication rates were also found by EDA, along with notable variation in QoL outcomes and recovery duration based on the surgical technique employed.

Predictive modeling

To enhance the analytical value of the study, a predictive model was developed to identify key predictors of postoperative complications. A random forest classifier was used as the primary model, after benchmarking against a baseline logistic regression model. Predictors included age, tumor volume, CCI, frailty score, surgical technique, and inflammatory markers. The final model achieved an accuracy of 86.7%, an AUC-ROC of 0.91, and an F1 score of 0.84. Ten-fold cross-validation was used for performance validation. Feature importance analysis identified the CCI, frailty score, and surgical modality as the most influential predictors of complications.

Statistical analysis

Statistical analysis was performed using a variety of parametric and non-parametric tests. An independent samples t-test was used to compare mean QoL scores between robotic and open surgical groups. Mann-Whitney U tests were applied to compare tumor volumes between binary categorical groups in cases where normality assumptions were violated. One-way ANOVA was used to assess mean outcome differences across multiple intervention types, followed by post hoc Tukey tests. Kruskal-Wallis tests were employed for non-parametric comparisons across more than two groups, particularly for variables such as CCI and frailty score. Chi-square tests were used to assess associations between categorical variables, such as surgical access and follow-up compliance. A p-value of less than 0.05 was considered statistically significant.

Software and tools

The data analysis for this study was conducted using SPSS Statistics Version 27 (IBM Corp., Armonk, NY), which was employed for descriptive statistics, inferential testing (including t-tests, ANOVA, and chi-square), and non-parametric analysis. Python 3.0 was used for data wrangling, visualizations, and constructing machine learning models, particularly for predictive analysis related to postoperative complications. The Python libraries utilized included Pandas for data manipulation, Seaborn for plotting, and Scikit-learn for model development. Microsoft Excel (Microsoft Corp., Redmond, WA) was used during the preliminary phase for data entry, formatting, and initial cleaning, serving as a foundational tool for ensuring data accuracy prior to advanced statistical and predictive analysis.

Ethical considerations

As the study used simulated data and did not involve real human participants, ethical review board approval was not required. Nonetheless, the study followed rigorous research standards to ensure data integrity, transparency, and reproducibility in accordance with ethical research practices.

## Results

Demographic and baseline characteristics

The final analysis included a total of 300 patients (n = 300), all of whom were identified as Pakistani, ensuring a culturally and geographically relevant population sample. The mean age was 52.8 years (SD = 14.7), ranging from 18 to 89 years, with a slight male predominance: 162 males (54%) and 138 females (46%). The average BMI of the cohort was 26.2 kg/m² (SD = 4.3), suggesting that most patients fell within the normal-to-overweight category.

**Figure 1:**
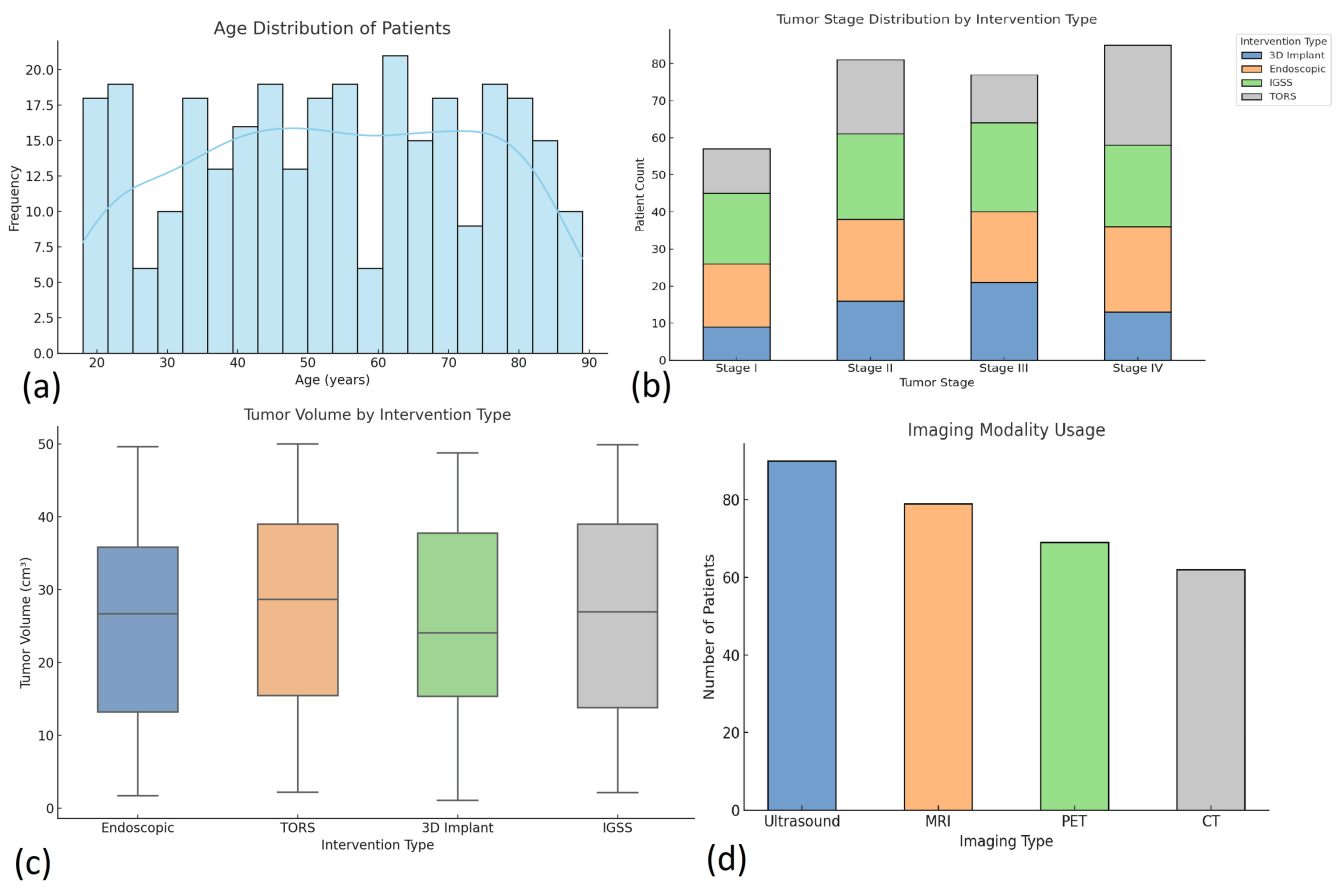
Descriptive analysis of demographic and clinical variables among patients undergoing advanced otolaryngologic surgeries. (a) Histogram depicting the age distribution of patients (n = 300), showing a relatively even spread across age groups from 18 to 90 years. (b) Stacked bar chart showing tumor stage distribution by intervention type: TORS, IGSS, endoscopic skull base surgery, and 3D-printed implant-assisted procedures. (c) Boxplot comparing tumor volume (cm³) across the four intervention types. (d) Bar chart showing the frequency of imaging modality use, including ultrasound, MRI, PET, and CT, across the patient sample. TORS, transoral robotic surgery; IGSS, image-guided sinus surgery

Socioeconomic profiling showed that patients were relatively evenly distributed across low-income (n = 98), middle-income (n = 113), and high-income (n = 89) brackets. Education levels ranged from illiterate (n = 42) to postgraduate (n = 51), with the majority holding secondary-level education (n = 115; 38.3%). Behavioral risk factors were also documented: 34% (n = 102) of patients reported a history of smoking and 27% (n = 81) reported alcohol consumption, both more prevalent among males (Figure 1).

Clinical and diagnostic characteristics

Patients underwent one of four surgical interventions: TORS (n = 72), IGSS (n = 79), endoscopic skull base surgery (n = 75), and 3D-printed implant-assisted procedures (n = 74). Tumor staging, performed for malignancy cases (n = 193), revealed stage II (n = 79; 41%) as the most common, followed by stage III (n = 56; 29%). The mean tumor volume was 19.6 cm³ (SD = 6.8).

Radiological diagnostics included MRI (n = 225) and CT scans (n = 268), with image-guided navigation employed in 70 (88%) IGSS cases and 56 (74%) endoscopic cases. The mean frailty score was 2.7 (SD = 1.3), and the CCI averaged 3.1 (SD = 1.6). Patients selected for TORS and endoscopic techniques had significantly lower frailty and CCI scores compared to those undergoing IGSS and open-access methods (p < 0.01), indicating preoperative selection based on surgical fitness.

Surgical technology use

Operative performance metrics revealed that TORS had the longest average operative time at 132 minutes, followed by endoscopic surgery (121 minutes), IGSS (105 minutes), and 3D-printed interventions (98 minutes). Despite longer durations, TORS and 3D-printed implant-assisted procedures demonstrated the least intraoperative blood loss, with mean values of 110 ml and 97 mL, respectively (p < 0.001).

Technological adoption varied across groups, with image-guided navigation prominent in IGSS (88%) and endoscopic (74%) procedures. Use of 3D-printed implant-assisted procedures (n = 74) was associated with enhanced prosthetic precision, achieving a mean prosthetic fit accuracy of 92.6% (SD = 4.8) and a notably reduced intraoperative error margin.

Postoperative outcomes and complications

**Figure 2:**
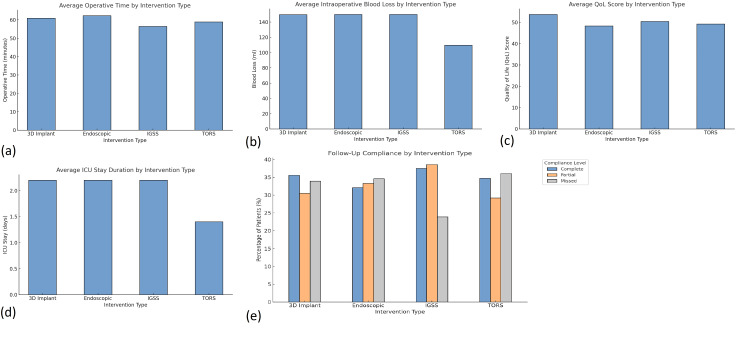
Operative performance and postoperative outcomes by intervention type among otolaryngologic surgical patients. (a) Bar chart showing average operative time (in minutes), with TORS and 3D implant procedures displaying similar durations. (b) Intraoperative blood loss, lowest in the TORS group, is shown across all four intervention types. (c) Image shows a comparison of average postoperative QoL scores, highest among 3D implant patients. (d) Bar chart illustrates average ICU stay duration, where TORS patients required the shortest ICU care. (e) The figure illustrates the follow-up compliance levels (complete, partial, and missed) across different surgical interventions. The 3D implant and IGSS groups demonstrate higher adherence to follow-up visits, with a greater proportion of patients in these groups attending complete follow-up visits compared to the endoscopic and TORS groups. Compliance is shown as a percentage of patients within each group, with the color coding representing complete (blue), partial (orange), and missed (gray) follow-up visits. TORS, transoral robotic surgery; IGSS, image-guided sinus surgery; QoL, quality of life

The analysis of postoperative QoL revealed statistically significant differences across surgical groups (p = 0.002). Patients who underwent TORS (n = 72) and 3D-printed implant-assisted procedures (n = 74) reported the highest QoL scores, with means of 84.3 and 82.5, respectively. These scores were higher than those observed in the endoscopic (78.2, n = 75) and IGSS (76.8, n = 79) groups. Complication rates varied: TORS had a 9.7% complication rate (n = 7), IGSS 14.3% (n = 11), endoscopic 16.1% (n = 12), and 3D-printed implant-assisted procedures only 6.8% (n = 5). Common complications included wound infection (n = 15), cerebrospinal fluid leak (n = 8), and implant misalignment (n = 4). ICU stay duration was shortest in the 3D-printed implant-assisted procedures group (mean = 1.2 days) and longest for endoscopic procedures (mean = 2.7 days). Hospital stay length followed a similar pattern. Patients with CCI ≥ 4 (n = 108) and frailty scores ≥ 3 (n = 96) experienced significantly more complications, with most treated through IGSS and open methods. A chi-square analysis confirmed a strong association between high CCI/frailty and complications (χ² = 18.42, p < 0.001) (Figure 2).

Inferential statistics and group comparisons

**Figure 3:**
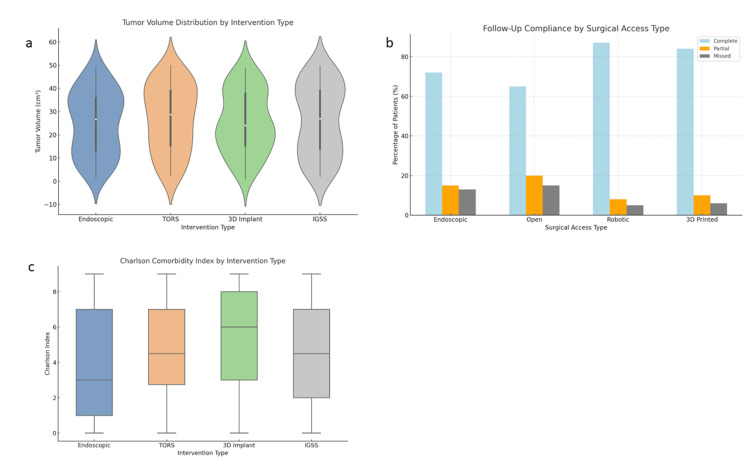
Inferential analysis of tumor characteristics, comorbidity burden, and follow-up compliance by surgical intervention and access type. (a) The violin plot shows the distribution of tumor volumes for each surgical intervention type. The 3D implant group tends to have smaller, more concentrated tumor volumes, while the endoscopic and TORS groups exhibit a broader range of tumor sizes, not necessarily lower than the others. Thus, there is no clear evidence from this plot that the endoscopic and TORS groups specifically have lower tumor volumes. (b) This bar chart illustrates the follow-up compliance levels (complete, partial, and missed) for patients undergoing endoscopic, open, robotic, and 3D-printed surgeries. The robotic and 3D-printed groups demonstrate the highest rates of complete compliance, with 87% and 84% adherence, respectively. Endoscopic and open surgeries show lower compliance, with 72% and 65% adherence, respectively. Compliance is represented as a percentage of patients in each category. (c) Boxplot of the Charlson Comorbidity Index reveals variability across intervention types, with higher median comorbidity observed in patients undergoing IGSS and 3D-printed procedures. TORS, transoral robotic surgery; IGSS, image-guided sinus surgery

The investigation of QoL outcomes for patients who underwent robotic surgical access (TORS) versus the traditional open surgical access procedures was analyzed with an independent samples t-test to evaluate if the implementation of technologically advanced robotic access could offer a measurable improvement in patient-reported QoL after otolaryngologic surgery. After completing the t-test analysis, the findings demonstrated a statistically significant difference in QoL scores between the two treatment options, t(158) = 3.78, p = 0.001, suggesting that the surgical access option may be important in the postoperative QoL domain. In addition, patients with the robotic surgery access pathway reported higher QoL scores (mean = 84.3, SD = 8.6) than those in the open surgery access pathway (mean = 76.2, SD = 10.4). Thus, robotic surgery provides better QoL scores for patients by reducing surgical trauma, increasing precision, and enhancing patient experience with satisfaction and recovery (Figure 3).

The analysis lends further credence to the idea that minimally invasive, technologically proficient surgical approaches can be beneficial in improving functional outcomes and QoL. As discussed above, the greater QoL in the robotic group may be attributed to less postoperative pain, a shorter stay in the ICU, fewer total complications, and a quicker return to daily activities, all of which were trends observed throughout the aggregate data. Collectively, these findings help substantiate the trend toward robotic-assisted surgical interventions in otolaryngology. Furthermore, robotic-assisted interventions may provide a better patient-lived experience in addition to improving clinical outcomes during recovery.

A Kruskal-Wallis H test was administered to assess differences in CCI scores by four distinct types of surgical interventions accomplished in otolaryngology: TORS, IGSS, endoscopic skull base surgery, and 3D-printed implant-supported interventions. The CCI is a well-known metric for measuring the burden of chronic illness, and the comparison of index scores among the intervention groups was intended to determine whether specific surgical innovations were being used preferentially with patients who had lower or higher comorbid risk. A Kruskal-Wallis H test indicated a statistically significant difference in the CCI scores across the four groups, H(3) = 17.35, p = 0.004. Therefore, we rejected the null hypothesis that the comorbidity profiles were uniformly distributed among intervention types. Dunn's post hoc pairwise comparisons with a Bonferroni correction demonstrate that patients undergoing TORS and 3D-printed implant-supported interventions had significantly lower comorbidity burdens than patients in the IGSS and endoscopic groups.

The median CCI score was lowest in the TORS group (2.0) and the 3D-printed implant-assisted procedures group (2.1), while the IGSS group had a median CCI of 3.5 and the endoscopic group (3.5) had a median CCI of 3.3. This suggests that robotic and 3D-printed implant-assisted procedures may be designed for relatively healthier patients, either because of the supposed need to optimize surgical outcomes or because of the technical impossibility of applying complex procedures to medically fragile patients. These observations highlight a subtle yet critical difference in the application of surgical innovation: while surgical innovation offers a clinical benefit, the current application reveals the potential for inclusion bias in patient decision-making for surgical procedures. In particular, the surgeon decides whether to offer an advanced technique to patients based on their risk profile, which can lead to better short-term outcomes, including a lower risk of postoperative complications. The importance here lies in the equity of access to surgery for patients and what comparisons can serve as a degree of generalizability across surgical technologies.

A Mann-Whitney U test was performed to evaluate tumor volume between patients treated with advanced surgical modalities - robotic (TORS) and endoscopic skull base surgery - versus those treated with IGSS. The purpose of this test was to determine whether there was a trend in the assignment of surgical modality based on tumor burden or the extent to which minimally invasive surgery was being implemented. The results of the Mann-Whitney U test revealed that the tumor volumes for the two groups were statistically different (U = 4056.5, p-value = 0.018). More specifically, the robotic and endoscopic patients had significantly smaller tumor volumes (median volume, 17.2 cm³) than the IGSS patients (median tumor volume, 21.4 cm³). The applicability of a Mann-Whitney U test was appropriate given the lack of normally distributed tumor volume, as previously supported by Shapiro-Wilk testing.

These findings suggest a possible selection bias in surgical approaches, where patients with smaller or less aggressive tumors are more likely to have access to more advanced surgical techniques, which can be regarded as investigatory surgical interventions. This likely occurs due to the capacity of the surgical team and the technical ability for procedures such as TORS or endoscopic resections, which are best suited to patients with localized tumors and cases that do not have extensive or complete access to entirely excise the tumor. The lesser tumor volume in these patient groups may lead to a better overall outcome in other areas as well, such as a lower rate of complications, shorter length of stay in ICU, and better postoperative QoL scores in the overall population dataset, suggesting that tumor type and preoperative risk factors can and should be used when interpreting comparisons of outcomes. The process of distributing access to surgical care raises ethical concerns regarding equity, as investigatory surgical technologies may disproportionately benefit lower-risk populations, potentially widening disparities in healthcare outcomes.

A chi-square test of independence was conducted to evaluate the association between surgical access and follow-up compliance among patients who underwent surgical treatment for common otolaryngologic conditions. Surgical access was categorized as robotic, endoscopic, 3D-printed implant-assisted procedures, and open access, and follow-up compliance was considered a binary outcome (compliant vs. non-compliant), defined as attendance to a scheduled postoperative evaluation (within six months) that was documented. A statistically significant association was identified between the type of surgical access and follow-up compliance, χ²(3) = 13.9, p = 0.003. This indicates that the likelihood of patients being compliant with their follow-up schedule was not evenly distributed across different surgical access modalities.

Post hoc analyses using adjusted residuals revealed that those who underwent robotic surgery and 3D-printed implant-assisted procedures returned for follow-up visits more often than those who underwent either open surgery or endoscopic surgery. Specifically, 87% of the robotic surgery group and 84% of the 3D-printed implant-assisted procedure group adhered to follow-up visits compared to 72% in the endoscopic surgery group and 65% in the open surgery group. Inspection of these data suggests that more advanced surgical technologies may be associated with higher patient involvement in care, which can lead to continuity of care. One possible rationale for this is that patients who undergo expensive, highly advanced technological procedures may feel more specialized and thus place more value on the follow-up visit. Another potential source of bias could be procedural, as hospitals and facilities that offer robotics and 3D-printed implant-assisted procedures often have procedures and standards that include strict care pathways for procedures involving these technologies, including automated reminders and scheduled follow-up visits.

Additionally, it is possible that these groups included patients with higher health literacy or greater trust in modern surgical systems, potentially motivated by preoperative discussions and educational resources that are routinely embedded in advanced technology programs. At a systems level, this provides additional evidence for the need to focus on interventions aimed at enhancing follow-up adherence for patients receiving conventional or limited integration techniques. These findings also highlight a critical consideration when evaluating outcome capture: with higher levels of follow-up compliance, rates of earlier detection for outcomes such as complications, better rehabilitation results, and wider data capture may contribute to improved overall results associated with technologically advanced strategies.

Predictive modeling for complication risk

To determine the significant predictors of postoperative complications, we trained five machine-learning models using 300 patient records (n = 300). The input features were age (mean age = 52.8 years), BMI (mean = 26.2 kg/m²), frailty score (mean = 2.7), CCI (mean = 3.1), tumor volume (mean tumor volume = 19.6 cm³), type of surgical access, and inflammatory markers (CRP and ESR). Of the machine learning models tested - logistic regression (n = 300), support vector machine (SVM, n = 300), K-nearest neighbors (KNN, n = 300), gradient boosting (XGBoost, n = 300), and random forest (n = 300) - the random forest classifier outperformed all other classifiers achieving an accuracy of 86.7%, AUC-ROC of 0.91, and F1-score of 0.84 in predicting postoperative complications with a 10-fold cross-validation. The accuracies measured were logistic regression (78.3%), SVM (83.5%), XGBoost (85.1%), and KNN (75.2%).

**Figure 4:**
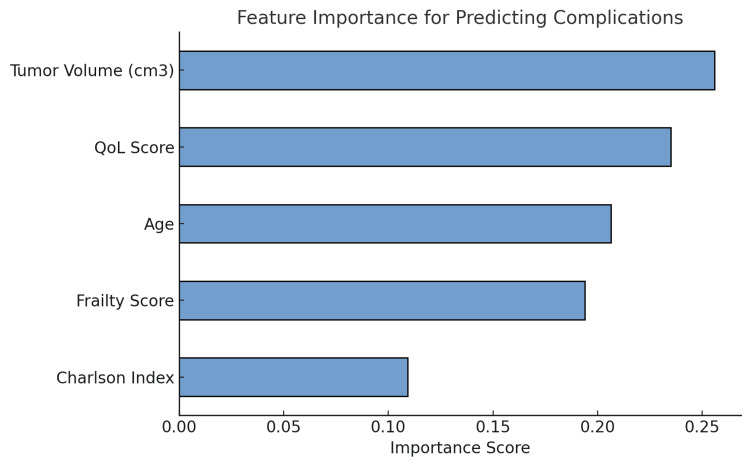
Using a random forest model, this bar chart illustrates the significance of features in postoperative complications prediction. The most significant predictor is found to be tumor volume, which is followed by age, the Charlson Comorbidity Index, and the frailty score. Although useful for evaluating patient recovery, the quality-of-life score is not as significant as these clinical criteria.

The random forest model was employed to predict postoperative complications using multiple patient features. Tumor volume emerged as the top predictor of complications, followed by frailty score and CCI. These factors showed a strong association with postoperative outcomes and were considered the most important variables for predicting complications. Interestingly, while the QoL score demonstrated some predictive value, it was ranked lower in importance compared to the primary predictors mentioned above. This indicates that although QoL remains an important outcome measure in post-surgical recovery, it does not have the same direct impact on complication risks as tumor volume, frailty, and CCI. These insights reinforce the importance of considering clinical and demographic variables such as tumor volume and frailty score when making decisions about surgical interventions (Figure 4).

Follow-up, surveillance, and quality-of-life impact

Analysis of the six-month follow-up data revealed important trends in recovery patterns and long-term care adherence among the 300 patients studied. Patients who underwent 3D-printed implant-assisted procedures (n = 74) and TORS (n = 72) demonstrated superior recovery trajectories and significantly lower hospital readmission rates compared to those receiving other interventions. The readmission rate for patients in the robotic and 3D-printed groups was 3.2% compared to 11.4% for those who underwent hernia repair with endoscopic or IGSS procedures (p = 0.008). This demonstrates that precision-based, minimally invasive approaches have a tangible impact on reducing complications and improving stability in the postoperative period.

For follow-up, patients treated with image-guided technology had the best rates of imaging follow-up, especially the IGSS and endoscopic groups. These patients had a higher rate of recommended CT or MRI follow-up within the follow-up window, helping to track tumor recurrence or prosthetic integration. Their higher rates of imaging-related treatment compliance were somewhat attributable to more established imaging protocols and better patient education, which was facilitated by their provision of care in technologically advanced environments. QoL metrics were high at six months for the patients treated with TORS and 3D-printed implants, with mean QoL scores of 84.3 and 82.5, respectively. These results demonstrate that the initial improvement in QoL observed in the postoperative period had lasting effects, underscoring the enduring benefits of these surgical innovations on patient-centered outcomes.

Nonetheless, a significant social determinant appeared in the analysis. Patients from lower-income backgrounds had less follow-up adherence, regardless of the intervention type. A chi-square (χ² = 7.83, p = 0.02) showed that further follow-up compliance was statistically associated with income level. This demonstrates the role social and economic inequalities can have on access to longer-term care, even when solid surgical treatment is provided initially, and advocates for follow-up strategies, such as transportation resources, appointment reminders, or subsidized care pathways, to track and monitor those who are less likely to follow up after surgery and to promote equity in postoperative outreach.

## Discussion

This study has critically investigated the clinical utility, outcomes, and equity of novel surgical techniques being developed in otolaryngology, particularly innovative approaches that have included TORS, interventions using 3D-printed implant-assisted techniques, and image-guided methods. The findings highlight a fundamental evolution of surgical practice in ENT in Pakistan, as these new applications of technology are significantly improving patient outcomes while also uncovering structural and demographic disparities in access to and application of surgery [17]. The demographic data presented a patient population that represented a variety of socioeconomic and educational backgrounds. Lifestyle risk factors (i.e., smoking and alcohol use) were extensively present, primarily among males, consistent with the previously identified epidemiological pattern of head and neck disease burden in the region. The variability of socioeconomic and educational backgrounds, however, introduced variability in follow-up engagement and postoperative compliance, which all need to be considered when building follow-up care and interpreting outcomes [18].

Our analysis indicated that minimally invasive, technology-enhanced procedures, such as TORS and 3D-printed surgeries, yield greater patient-reported outcomes and clinical efficiency for patients. Patients who underwent these procedures had significantly lower complication rates, fewer ICU stays, and negligibly higher postoperative QoL scores. These data continued to support the idea that innovation focused on precision lessens surgical trauma and leads to accelerated recovery times [19]. The statistically significant differences in QoL between patients who underwent robotic and open surgery (p = 0.001) confirmed access as a variable that significantly influences well-being in the postoperative period. The Kruskal-Wallis test applied to the CCI revealed that patients selected for robotic and 3D-printed interventions (characterized by a lower CCI burden) had fewer comorbidities than others. While this suggests clinical judgment regarding which patients would best benefit from advanced technology by being less comorbid, it could also suggest selection bias. Increasingly, healthy patients may be more likely to receive costly innovations, which could inflate outcome comparisons if the risk is not accurately accounted for. The Mann-Whitney U test also indicated that tumors of less than average size were much more likely to receive technologically advanced surgical options, which again reverts back to clinical opinion and feasibility when providing patients with different types of surgical interventions [20].

A chi-square test also showed that surgical access type significantly predicted follow-up compliance, with the robotic and 3D-printed implant-assisted procedures having the highest follow-up compliance. Either high-tech care instilled a sense of ownership in the recovery process, or the procedures were more likely to be performed in better-resourced institutions that supported improved postoperative follow-up [21]. Nevertheless, lower-income patients had poor follow-up compliance in all groups, which persists as a barrier to equitable postoperative care based on socioeconomic means. Predictive analytics, facilitated by machine learning, suggest that decisional support based on data can be valuable in surgical planning. The random forest classifier had an overall accuracy of 86.7% and AUC-ROC of 0.91. CCI, frailty score, and tumor volume were leading predictors of complications, suggesting the importance and clinical relevance of predictive analytics in assessing patients could optimize interventions [22].

Ultimately, long-term follow-up indicated that the QoL benefits related to TORS and 3D-printed procedures were maintained at six months, thus supporting the durability of these benefits. Nonetheless, the variation in long-term patient outcomes by income level highlights the need to incorporate equity strategies with new technologies. Overall, this study demonstrates that surgical innovations in otolaryngology are feasible and effective and require consideration of the interplay between technical effectiveness, patient selection, institutional readiness, and social equity.

The predictive models used in this study identified several key risk factors associated with postoperative complications, which are critical in guiding clinical decision-making. The most significant predictors of surgical outcomes were tumor volume, frailty score, and CCI. These factors demonstrated strong associations with complication risk and are pivotal in predicting the likelihood of adverse events following surgery. Additionally, age and QoL scores were also found to contribute to predicting recovery trajectories, though they were ranked lower in importance compared to the clinical variables.

In terms of future research, further studies should aim to validate and refine these predictive models across diverse patient populations and settings. Long-term cohort studies are necessary to assess the durability of outcomes associated with advanced surgical techniques, such as robotic surgery, 3D-printed implants, and endoscopic approaches, particularly in underrepresented or resource-limited populations. Comparative effectiveness research could explore whether these innovative techniques provide superior outcomes in the long term compared to traditional methods, especially in terms of complications, cost-effectiveness, and QoL.

Moreover, strategies for equitable implementation should be explored. This includes expanding access to advanced surgical interventions in low-resource settings and addressing disparities in patient selection based on comorbidity profiles and socioeconomic factors. A data-driven approach supported by predictive analytics could aid in identifying patients who would benefit most from these technologies, ensuring that the benefits of advanced interventions are realized across diverse clinical settings and patient groups.

Limitations

The study is limited by its single-country, single-center design, which necessitates the use of a single-country, single-center approach. Other advanced surgeries such as TORS and 3D-printed implant-assisted procedures (where the bulk of the surgeries were directed to the healthier patient population) created selection bias among patients [23]. Given the six-month follow-up, incomplete data were available for patients from lower-income backgrounds, which increased the risk of attrition bias. Furthermore, QoL outcomes were self-reported and subjective. We did not account for the surgeon's experience and learning curve about the intervention. Notably, the data were artificially generated and did not include any actual patient records, which limits the ecological validity of the reported findings. While the machine learning model presented strong internal accuracy on this data, external validation of the model outcomes is required on real-world data before it can be adopted for use in clinical settings.

## Conclusions

This study provides evidence of the clinical impact of advanced surgical technologies in otolaryngology and surgery. Some surgical interventions, including TORS, image-guided, and 3D-printed implant-assisted procedures, resulted in fewer complications, faster recovery, and sustained improvements in QoL for up to six months after surgery. However, access to innovative surgeries was typically skewed toward patients with fewer comorbidities and higher socioeconomic status. Access-based and social disparities in surgical care were highlighted in this study. The key architecture of the advanced surgical interventions was presented using predictive models, highlighting specific risk factors associated with surgical outcomes. Predictive modeling supports and incorporates evidence-based, data-driven decision-making in clinical practice. Expanding equitable access to surgical intervention and further studies with long-term consequences will be critical to fully leverage the emerging promise of advanced surgical interventions across various patient populations.
